# Oxidative stress and alopecia areata

**Published:** 2015

**Authors:** BE Prie, VM Voiculescu, OB Ionescu-Bozdog, B Petrutescu, L Iosif, LE Gaman, VG Clatici, I Stoian, C Giurcaneanu

**Affiliations:** * ”Carol Davila” University of Medicine and Pharmacy, Bucharest, Romania; ** “Elias” University Emergency Hospital, Bucharest, Romania; *** Polimed Clinic, Bucharest, Romania; **** Sanmed Medical Center, Bucharest, Romania; ***** R&D Irist Labmed, Bucharest, Romania

**Keywords:** alopecia areata, oxidative stress, antioxidants

## Abstract

Alopecia areata (AA) is an inflammatory and autoimmune disease presenting with non-scarring hair loss. The aethiopathogenesis of alopecia areata is unclear and many factors including autoimmunity, genetic predisposition, emotional and environmental stress are thought to play important roles in its development. Antioxidant/ oxidant balance perturbation is a common feature in autoimmune, emotional and environmental stress. Therefore, our paper discusses the implications of oxidative stress in alopecia areata.

**Abbreviations:** AA = alopecia areata, ROS = reactive oxygen species, H2O2 = hydrogen peroxide, TBARS = thiobarbituric acid rective substances, MDA = malondialdehyde, TBARS = thiobarbituric acid-reactive substances, SOD = superoxide dismutase, CAT = catalase, GSH-Px = glutathione peroxidase, PON1 = paraoxonase 1, HO-1 = hemoxigenase 1, TrxR = thioredoxin reductase, GSH = glutathione

## Introduction

Alopecia areata (AA) is a chronic, inflammatory and autoimmune disease, presenting with non-scarring hair loss [**1**]. AA is a disorder with different clinical presentations. It most commonly affects the scalp, but any hair bearing area of the skin can be affected [**1**]. The hair loss presents as circumscribed patches (most common), progression of hair loss and involvement of the entire scalp (alopecia totalis), or the involvement of the entire body (alopecia universalis) [**2**]. Histologically, AA is an autoimmune disorder with autoaggresive T cells directed against the anagen hair follicles [**3**,**4**]. In the acute stages, there are abundant inflammatory infiltrates that surround hair follicles, but the bulb retains its capacity for growth and follicular stem cells remain viable [**4**]. The aethiopathogenesis of AA is unclear and many factors including the patient’s genetic constitution, the atopic state, emotional and environmental stress are claimed to be involved in its development [**1**,**5**,**6**]

Antioxidant/ oxidant balance perturbation is a common feature in autoimmune, emotional and environmental stress. Therefore, our paper discusses the implications of oxidative stress in AA. 

## Reactive oxygen species (ROS) and antioxidant systems

Oxidative stress occurs as a result of inadequate antioxidant defense or overproduction of free radicals. Its presence has been shown in many dermatological diseases including psoriasis [**7**], vitiligo [**8**], atopic dermatitis, lichen planus [**9**], acne vulgaris [**10**], pemfigus vulgaris [**11**], seborrheic dermatitis [**12**,**13**], skin cancers [**14**]. The skin is chronically exposed to both endogenous and environmental pro-oxidant agents leading to the generation of ROS, which are involved in the damage of cellular constituents such as nucleic acids, proteins and cell membrane lipids [**15**,**16**]. 

As a result of permanent oxidative processes, the body has developed antioxidant defense mechanisms to prevent the attack of biological molecules [**17**,**18**] 

## Evidence of lipid peroxidation presence in AA

Lipid peroxidation represents the hallmark of oxidative stress, which results after cell membrane lipids are exposed to ROS [**17**,**18**]. Lipid peroxides and their breaking-down products such as malondialdehyde (MDA) can affect the normal function of most mammalian cells [**15**] and their level correlates with the degree of lipid peroxidation [**18**].

Several studies evaluated the levels of MDA in plasma and erythrocytes, but also in scalp biopsies of patients with AA. Naziroglu et al. found significantly higher TBARS levels in plasma and erythrocytes of patients with alopecia than in controls [**19**]. Akar et al. also found significantly higher TBARS levels in tissues from scalp biopsies of patients with AA compared to healthy subjects and two times higher levels of TBARS in early phase than late phase of the disease [**20**]. Koca et al. supported previous findings and indicated higher levels of serum MDA in patients with AA compared with control subjects [**18**]. Abdel Fattah et al. reported increased levels of MDA in plasma and tissues. He also noted that the more severe (polyAA, alopecia totalis, alopecia universalis) and the longer (≥6 months) the disease, the higher the levels of MDA [**21**,**22**]. Yenin et al. and Bakry et al. also reported significantly higher levels of MDA in plasma patients with AA compared with control subjects [**17**,**23**].

## Enzymatic antioxidants in alopecia areata

Superoxide dismutase (SOD) is the prime antioxidant enzyme against damage caused by superoxide anion, converting it to oxygen and hydrogen peroxide (H2O2) [**24**,**25**]. The dysfunction of SOD was documented in patients with AA [**17**,**18**,**20**,**21**] and also that, it can become antigenic if continuously exposed to ROS and nitrogen species [**25**]. Akar et al. found elevated levels of SOD in tissues from scalp biopsies of patients with AA versus control subjects and two times increased SOD in early versus late phase of the disease [**19**]. Abdel Fattah et al. observed decreased SOD activities and noted that the more severe (polyAA, alopecia totalis and alopecia universalis) and the longer (≥ 6 months) the disease, the lower the levels of SOD [**21**]. Koca et al. and Yenin et al. also found decreased SOD activities in serum and erythrocytes of patients with AA versus control subjects [**18**]. Rasheed et al. reported that nitric oxide-damaged erythrocyte SOD initiate autoantibodies in patients with AA and also that it may be an important biomarker for disease progression [**25**].

Catalase (CAT) inactivates H2O2 via its dissociation to water and oxygen. Yenin et al. have not noticed any differences between erythrocyte CAT of patients with AA versus control subjects [**17**].

Glutathione peroxidase (GSH-Px) can neutralize a broad range of peroxides. In the presence of glutathione, it also converts H2O2 to water and oxygen [**26**]. Naziroglu et al. reported that plasma and erythrocyte GSH-Px was reduced in patients with AA versus controls [**19**]. 

Akar et al. reported a two times increased GSH-Px activity in tissues from scalp biopsies of patients with AA than in controls [**20**]. Yenin et al. noted that erythrocyte GSH-Px activity was significantly reduced in patients with AA versus control subjects [**17**].

Paraoxonase 1 (PON 1) is a Ca-dependent serum esterase associated with HDL and contributes to antiatherogenic effects of HDL [**27**,**28**]. Lower paraoxonase activity has been found in serum and tissues of patients with alopecia areata. 

Hemoxigenase-1 (HO-1) is the rate-limiting enzyme implicated in catabolism of hem with the release of free irons, carbon monoxide and biliverdin, which is reduced to bilirubin with antioxidant effects [**29**]. It has an important cytoprotective and antioxidant role, limiting skin inflammation in T cell-dependent inflammatory disorders and suppressing of antigen presenting cells [**30**,**31**]. HO-1 expression was significantly decreased in scalp biopsies of patients with AA than in control subjects [**29**].

Thioredoxin reductase (TrxR) is a selenoenzyme that exerts diverse cellular functions, by reduction of oxidized thioredoxin in the presence of NADPH [**32**,**33**]. Reduced thioredoxin serves as an electron donor for thioredoxin peroxidase, which reduces H2O2 to water [**33**]. The thioredoxin/ thioredoxin reductase system removes free radicals [**34**] and the perturbation in TrxR activity appears in many immunological diseases or certain malignancies [**35**]. Sohn et al. reported that the decrease of TrxR1 may be a cause for glucocorticoid resistance in AA [**32**].

**Non- enzymatic antioxidants in alopecia areata**

Glutathione is a reducing agent with the ability to neutralize ROS [**36**]. It is also a cofactor for antioxidant enzymes [**22**]. Naziroglu et al. reported decreased levels of GSH in plasma and erythrocytes of AA patients, compared with controls [**19**].

Vitamin E and beta-carotene are essential fat-soluble vitamins and protectors of the cell membranes against lipid peroxidation [**19**], interacting preferentially with free radicals such as lipid peroxyl radicals [**15**]. Naziroglu et al. reported significant decrease levels of beta-carotene in plasma and erythrocytes of AA patients than in controls, but not a statistically significant degree of vitamin E [**19**]. Ramadan et al. also noted lower tissue and serum vitamine E in AA patients than in controls. 

**AA therapy and oxidative stress**

Medications for AA are still under development. Treatments thought to reduce oxidative stress are under consideration based on research studies presenting evidence of the presence of oxidative stress in alopecia areata. Administration of a substrate for paraoxonase N-(3-oxododecanoyl)-L-homoserine-lactone decreased oxidative stress and stimulated hair growth in ob/ ob mice [**37**]. Tempol, a synthetic permeable SOD mimetic normalized hair growth in a mouse model of chronic restraint stress [**38**]. Tocotrienols, antioxidants from vitamin E, increased hair numbers in volunteers with hair loss [**39**]. Interestingly, anthralin, currently used in alopecia areata treatment exerts its effect via the generation of free radicals [**40**]. 

## Conclusion

Although the etiology of AA is still unclear, there are studies that support the association between oxidative stress and AA [**41**]. The conflicting results observed in some studies can be explained by inclusions criteria and different stages of the disease considered. The attenuation of oxidative stress might be a relevant therapeutic approach and antioxidants can be recommended as additional drugs in AA treatment [**23**].

**Acknowledgement**

This paper is supported by the Sectorial Operational Programme Human Resources Development (SOP HRD), financed from the European Social Fund and by the Romanian Government under the contract number POSDRU/159/1.5/S/137390.

**Disclosures**

None
